# Robot‐assisted laparoscopic pyeloplasty for ureteropelvic junction obstruction due to aberrant blood vessel with ipsilateral retrocaval ureter

**DOI:** 10.1002/iju5.12304

**Published:** 2021-06-03

**Authors:** Yuta Inoue, Yasuyuki Naitoh, Jun Ajiki, Ayako Fukui, Takeshi Yamada, Atsuko Fujihara, Kaori Yamada, Fumiya Hongo, Osamu Ukimura

**Affiliations:** ^1^ Department of Urology Kyoto Prefectural University of Medicine Kyoto Japan; ^2^ Department of Diagnostic Radiology Kyoto First Red Cross Hospital Kyoto Japan

**Keywords:** dismembered pyeloplasty, retrocaval ureter, retrograde pyelography, robot‐assisted laparoscopic pyeloplasty, ureteropelvic junction obstruction

## Abstract

**Introduction:**

Ureteropelvic junction obstruction is a common congenital anomaly that causes hydronephrosis but rarely accompanies ipsilateral retrocaval ureter.

**Case presentation:**

A 39‐year‐old woman, who visited to our hospital complaining of worsened right low back pain and fever, was diagnosed with right hydronephrosis due to ureteropelvic junction obstruction by contrast‐enhanced computed tomography. Intraoperatively before the planned robot‐assisted laparoscopic pyeloplasty, retrograde pyelography was performed to reveal concomitant ipsilateral retrocaval ureter. Laparoscopically, ureteropelvic junction obstruction due to aberrant blood vessel and coexisting retrocaval ureter was confirmed. Transposition of the ureter from posterior to anterior of the inferior vena cava and following dismembered pyeloplasty was performed. Two years after surgery, her right hydronephrosis improved and she had no complain of any symptom.

**Conclusion:**

Retrocaval ureter is a rare abnormality; however, combination of preoperative retrograde pyelography and laparoscopic evaluation was important for management of this concomitant abnormality.

**Abbreviations**
CECT: contrast‐enhanced computed tomography
CT: computed tomography
IVC: inferior vena cava
MAG3: mercaptoacetyltriglycine
RALP: robot‐assisted laparoscopic pyeloplasty
RP: retrograde pyelography
UPJO: ureteropelvic junction obstruction

**Keynote message**

Ureteropelvic junction obstruction rarely accompanies ipsilateral retrocaval ureter, which could be detected by retrograde pyelography. It is important to consider potential concomitant ureteral anomaly such as retrocaval ureter before surgery, in order to manage the surgical strategy.

## Introduction

UPJO is a common congenital abnormality that causes hydronephrosis. Nearly 50% of UPJO have other congenital urinary tract malformations such as vesicoureteral reflux, ureterovesical junction obstruction, double renal pelvis and ureter, congenital midureteral stricture, and horseshoe kidney.^1^ But there was only one report of UPJO that has a single concomitance of retrocaval ureter.^2^ To our best knowledge, we hereby document a case report of these comorbidities secondary in the world and first in Japan.

## Case presentation

**Fig. 1:**
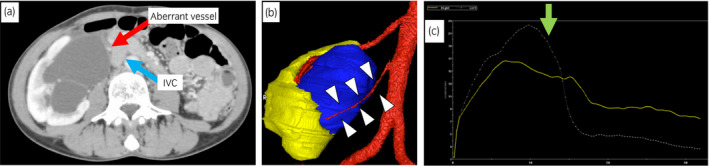
The CECT and Tc‐99m MAG3 renal scan findings. (a) Right hydronephrosis was observed, but the right ureter was not clearly identified in the CECT (probably because of little distal ureteral flow). Blue arrow indicates IVC, and red arrow indicates the aberrant vessel. (b) Reconstructed 3D image from CECT with Osirix^®^, imaging analysis software. An aberrant blood vessel that obstructs ureteropelvic junction was observed. White arrows suggest the running course of the aberrant blood vessel, whichwas considered a direct cause of UPJO. (c) Tc‐99m MAG3 renal scan finding. Yellow solid line represents the right kidney and white dotted line represents the left kidney. Green arrow shows administration of diuretics.

A 39‐year‐old woman visited to our hospital complaining of worsened right low back pain and fever in 1 month before her first visit. She had experienced the occasional similar pain for several years and had an experience of pyelonephritis 10 years ago. Her initial laboratory data had no abnormal findings. Her urine test was clear, and her urine cytology was negative. Ultrasound imaging revealed right gross hydronephrosis. CECT also detected right gross hydronephrosis, but the right ureter was unclear (Fig. 1a and Video S1). CECT was reconstructed to 3D imaging with Osirix^®^ (Pixmeo SARL, Geneva, Swiss), imaging analysis software, which found out an aberrant blood vessel contacting with the right ureteropelvic junction (Fig. 1b). According to these examinations, UPJO was preoperatively diagnosed due to the aberrant blood vessel. Tc‐99m MAG3 renal scan confirmed that the right kidney exhibited obstructive pattern and poor diuretic response (T1/2 of the left and right kidneys was 4.62 and 21.07 min, respectively) (Fig. 1c). The right kidney function was similarly preserved to the left kidney function (MAG3 clearance of the left and right kidneys was 184.9 and 163.7 ml/min/1.73 m^2^, respectively).

**Fig. 2:**
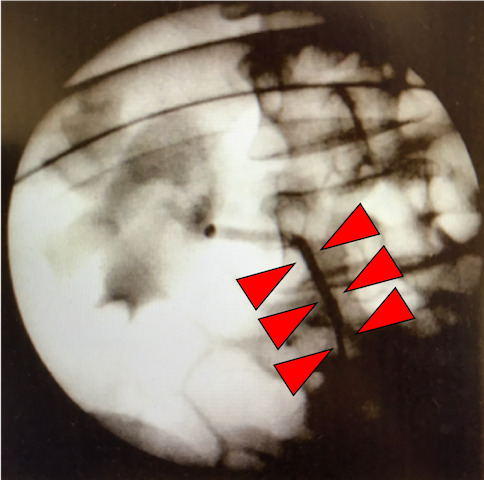
Preoperative RP. The right ureter shifted medial (red arrows) compared with the normal position

**Fig. 3:**
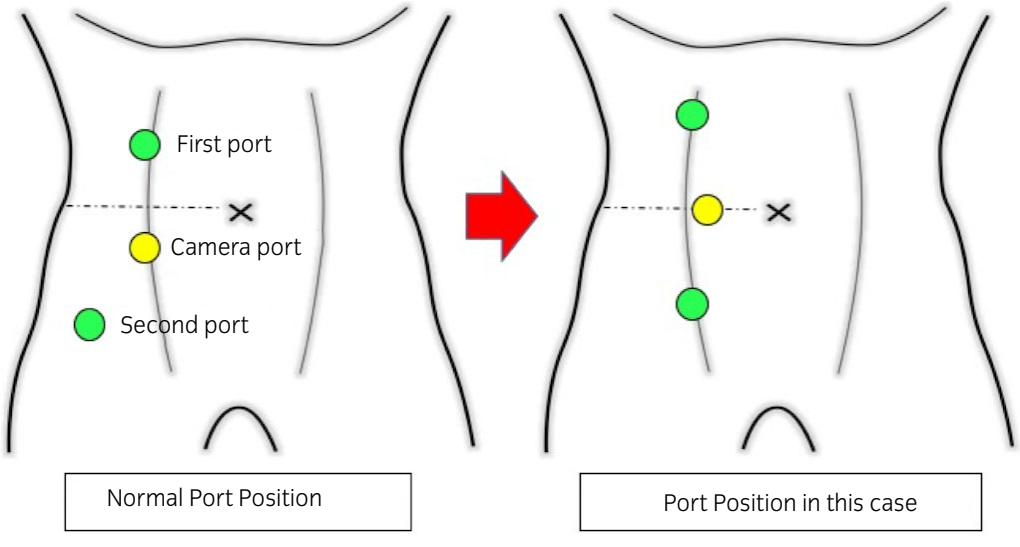
The scheme of the port position. The left image shows the normal port position for pyeloplasty in our institution, and the right image shows the port position of this case

**Fig. 4:**
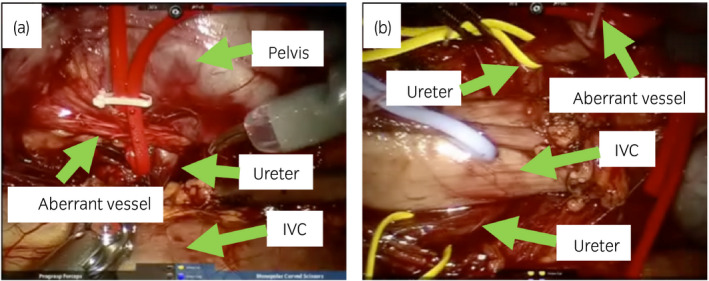
Intraoperative image of the RALP. The left image (a) shows that the right ureteropelvic junction was obstructed by an aberrant blood vessel as a direct cause of the hydronephrosis in UPJO. The right image (b) shows the laparoscopic confirmation of retrocaval ureter that the right ureter ran behind IVC

Surgical treatment was indicated because of the symptomatic disease. Preoperative RP showed that the right ureter shifted inward compared with the normal position, which suggested the coexistence of the retrocaval ureter (Fig. 2). RALP was conducted by a three‐port transperitoneal approach, with putting port position more medial than usual to facilitate detachment around the right ureter (Fig. 3). The swollen right renal pelvis and the aberrant blood vessel were identified (Fig. 4a). The right ureter was peeled downward and was confirmed to be positioned behind the IVC (Fig. 4b). The renal pelvis was dissected, and the ureter was repositioned anterior to the IVC. After removing the strictured ureteropelvic junction, the ureter and the renal pelvis were spatulated, inserted with 6‐Fr double‐J stent, and reanastomosed in a tension‐free fashion by interrupted suture technique with 5‐0 sutures; 10‐Fr BLAKE Silicon drain was put into the anastomosis place. Blood loss was uncountable. The operation time was 403 min, and the robot console time was 301 min. We represent edited surgical video in Video S1.

The urethral catheter and the drain were removed 5 and 6 days after surgery, respectively. No perioperative adverse events were found. The double‐J stent was removed 2 months after surgery. No recurrence of the symptoms was observed, and her right hydronephrosis improved in 2 years after surgery.

## Discussion

UPJO is a common congenital abnormality that causes hydronephrosis both in children and in adults. Adult symptomatic UPJO is generally caused by an aberrant blood vessel.^3^ Retrocaval ureter is a rare congenital abnormality, which causes ipsilateral hydronephrosis, which is often detected in the 30th or 40th year of life.^4^ Retrocaval ureter is a developmental anomaly of IVC, caused by the abnormal persistence of the right subcardinal vein, which is anterior to the right ureter.^4^ Around 20% of the retrocaval ureter accompanies the other congenital anomalies such as horseshoe kidney, undescended testis, or contralateral renal agenesis.^5^ Radiologically, retrocaval ureter is classified into Types 1 and 2; 90% of the retrocaval ureter is Type 1 and shows “S” shape or fishhook appearance. Type 2 retrocaval ureter is a rare subtype and shows sickle‐shape ureter.^6^

UPJO and retrocaval ureter are completely different anomalies, but they cause ipsilateral hydronephrosis in adult. Coexistence of UPJO and retrocaval ureter without other anomalies is very rare. To our best knowledge, there is only one report published by Fletcher and Lecky in 1971.^2^ Because the case reported by Fletcher and Lecky did not perform CT scan, we hereby present the first CECT finding of the coexistence of UPJO and retrocaval ureter. When retrocaval ureter was a single abnormality, CECT could detect retrocaval ureter.^7^, ^8^ In our case, unfortunately, the coexisting retrocaval ureter could not be diagnosed preoperatively by CECT. We supposed that the cause of hydronephrosis was UPJO because aberrant blood vessel is proximal to the retrocaval ureter. It is conceivable that CECT could not detect the distal ureteral anomaly, retrocaval ureter, because UPJO impaired the distal urine flow. In contrast, preoperative RP could show abnormal position of the right ureter, suggesting the presence of retrocaval ureter. Retrospectively, the results of our RP were similar to those of the case reported by Fletcher and Lecky.^2^ Especially in our case, intraoperative RP just before port placement helped us to assume this possible significant ureteral anomaly for the first time. This was important because the surgery could be managed by considering the presence of the retrocaval ureter, resulting in that the port positions could be positioned more medially than usual to allow us to manage retrocaval ureter easily during surgery.

The consensus about the surgery for retrocaval ureter is the same as that for UPJO, dismembered pyeloplasty.^9^ Briefly, after dismembering the renal pelvis and the ureteropelvic junction, the ureter was mobilized from the back to the front of IVC followed by reanastomosis of the renal pelvis and the ureter. Although there are few reports, RALP would also be a well applicable choice for retrocaval ureter.^10^, ^11^

In our case, the retrocaval segment, which was not a direct cause of hydronephrosis caused directly by aberrant blood vessel, was surgically shifted anterior to the IVC for preventing a future potential recurrence of hydronephrosis. Although this case was very rare, RALP was successfully completed by arrangement of the port position based on the findings of the preoperative RP. The possibility of concomitant abnormalities including retrocaval ureter in UPJO surgery should be considered.

## Conflict of interest

The authors declare no conflict of interest.

## Supporting information

**Video S1**. CECT finding. CECT also detected right gross hydronephrosis but the right ureter was unclear.

**Video S2**. Edited surgical video. An aberrant blood vessel obstructed ureteropelvic junction (0:00). The right ureter ran behind IVC (0:03). The renal pelvis was dissected, the ureter was repositioned anterior to the IVC and the strictured ureter was removed (0:12). The ureter and the renal pelvis were spatulated, inserted with 6‐Fr double‐J stent, and reanastomosed in a tension‐free fashion by interrupted suture technique with 5‐0 sutures (0:23).
